# Association between liver biomarkers and risk of cognitive impairment and dementia: A systematic review and meta-analysis

**DOI:** 10.12669/pjms.41.7.12321

**Published:** 2025-07

**Authors:** Ying Zhong, Lei Li

**Affiliations:** 3 Ying Zhong, Department of Geriatrics, Huzhou Third Municipal Hospital, the Affiliated Hospital of Huzhou University, Huzhou, Zhejiang Province 313000, P.R. China; 4 Lei Li, Department of Geriatrics, Huzhou Third Municipal Hospital, the Affiliated Hospital of Huzhou University, Huzhou, Zhejiang Province 313000, P.R. China

**Keywords:** Brain, Cognition, Elderly, Hepatic disease, Liver disease

## Abstract

**Objective:**

We aimed to systematically examine the literature and conduct a meta-analysis to assess if liver biomarkers like alanine aminotransferase (ALT), aspartate aminotransferase (AST), gamma-glutamyl transpeptidase (GGT) could be predictors of cognitive impairment and dementia.

**Methods:**

Online databases of Scopus, Embase, PubMed, CENTRAL, and Web of Science were searched for all observational studies examining the research question. The last date of the search was August 20, 2024.

**Results:**

We selected 13 studies. Pooled analysis showed that there was no significant association between ALT levels or AST levels and risk of cognitive impairment. Meta-analysis showed the higher AST: ALT ratio and higher GGT levels were associated with a statistically significant increased risk of cognitive impairment. Lower ALT levels were associated with a significant increase in the risk of dementia but no significant association was noted with AST or GGT levels.

**Conclusions:**

Preliminary evidence indicates that liver biomarkers may have a role in predicting the risk of cognitive impairment/dementia. A high AST: ALT ratio may be linked with an increased risk of cognitive impairment and dementia. Likewise, high GGT and low ALT levels seem to be associated with increased risk of cognitive impairment and dementia respectively.

***PROSPERO Registration Number:*** CRD42024579967.

## INTRODUCTION

Cognitive impairment and dementia account for a significant global public health burden affecting nearly 50 million individuals worldwide.^1^ Moreover, with increased life expectancy and an aging population, the incidence of neurological disorders is bound to rise dramatically in the coming decades. Trends indicate that the prevalence of dementia may increase exponentially resulting in about 106 million patients by 2050.^2^ Cognitive impairment lies in the spectrum between normal aging and dementia wherein there is memory loss more than expected for the given age but individuals do not meet the meet currently accepted criteria for dementia. It may therefore be considered as a preliminary stage of dementia with a high probability of conversion to clinical dementia as compared to age-matched adults.^3^ Both these conditions lead to a significant economic and emotional burden on the society as a whole and there is a need to limit their incidence by identifying relevant risk factors and taking preventive measures.^4^

Alzheimer’s disease (AD) is the commonest age-related dementia.^5^ While the majority risk of AD is dependent on genetic factors, several metabolic anomalies like chronic inflammation, insulin resistance, and oxidative stress are postulated to have a role in the pathogenesis of the disease.^3^ AD is considered a metabolic disorder with patients experiencing both central and peripheral metabolic dysfunction.^6^ The liver is central to the metabolic function of the body and its activity primarily determines the overall metabolic status of the body.^7^ Research shows that liver dysfunction may be one of the factors in the progression of AD. AD is characterized by synaptic and neuronal degeneration, amyloid plaques, and neurofibrillary tangles. The amyloid plaques are composed of amyloid-beta peptide (Aβ) and it is postulated that impaired clearance of the same by the liver could result in excessive deposition in the brain resulting in AD.^8^ There is also evidence indicating that individuals with liver diseases have changes in brain structure.^9^ Therefore, there may be a link between hepatic dysfunction and cognitive impairment and dementia which needs to be explored further.^10^

Hepatic function is commonly measured by assessing levels of liver enzymes like alanine aminotransferase (ALT), aspartate aminotransferase (AST), AST/ALT ratio, and gamma-glutamyl transferase (GGT) in the peripheral blood.^11^ High levels of these enzymes have been implicated in several metabolic and cardiovascular disorders^12^-^14^ which are themselves linked with increased risk of cognitive impairment and dementia.^3^ However, it is unclear if these markers could be predictors of cognitive impairment and dementia. While this has been examined by several studies in literature, to date, no systematic review has been attempted to collate such evidence. Hence, we present the result of the first systematic review and meta-analysis examining if liver enzymes could be potential biomarkers of cognitive impairment and dementia.

## METHODS

We searched PubMed, CENTRAL, Scopus, Embase, and Web of Science databases using free and MeSH keywords. The query used was: (Gamma-glutamyltransferase) OR (GGT) OR (gamma-glutamyl transpeptidase)) OR (alanine aminotransferase)) OR (ALT)) OR (aspartate aminotransferase)) OR (AST)) OR (liver enzyme) AND (cognition) OR (cognitive) OR (dementia) OR (Alzheimer’s disease). The query was customized as per the needs of each of the databases. The search was conducted independently by two reviewers (YZ & LL) in consultation with a medical librarian expert in literature search. The search was finalized on 20^th^ August 2024 and was restricted to publications in English only.

The articles found were merged in the reference manager software (EndNote) and deduplicated. Initial screening of titles and abstracts was done for all records. Studies found relevant by either reviewer underwent further screening by full-text reading. Two reviewers (YZ & LL) were independently involved in the final screening and all disagreements were resolved by consensus. We then also searched the reference lists of included studies and gray literature by Google Scholar for any missed articles.

### Inclusion criteria:

This PRISMA-compliant^15^ and PROSPERO-registered (CRD42024579967) systematic review and meta-analysis aimed to examine the clinical question: can liver markers namely ALT, AST, ALT: AST, and GGT predict the risk of cognitive impairment and dementia? Based on this the inclusion criteria were formulated by all reviewers by consensus before beginning the literature search. The authors decided to include all observational study designs enrolling the general population. Studies were to segregate the participants based on serum ALT, AST, ALT: AST, or GGT values at any cut-off deemed suitable. Studies were to assess the risk of cognitive impairment or dementia based on the level of these markers and report adjusted outcomes as effect size with 95% confidence intervals (CI). There was no limitation on the method of assessing cognitive impairment and dementia. Also, all causes of dementia were accepted for the review.

### Exclusion Criteria:

We excluded studies on specific disease populations like only diabetics or post-stroke patients. We restricted ourselves to complete articles published in peer-reviewed journals and did not include articles only as abstracts, theses, and in non-peer-reviewed journals or databases.

### Data extraction:

The same reviewers also extracted data from the studies independently of each other by using a pre-formulated table. Information on the study authors, publication details, study design, location, inclusion and exclusion criteria, sample size, age and gender of participants, liver marker used, cut-off of the same, outcome assessed and method of assessment, adjusted covariates, follow-up in case of cohort studies and effect size with 95% CI was extracted from each article. If any outcome data was missing or incomplete, the corresponding author was contacted once by the review authors by email. If any study still had missing outcome data, we excluded it from the meta-analysis.

### Risk of bias:

All included articles underwent quality assessment using the Newcastle-Ottawa Scale (NOS) to assess study quality.^16^ The questions of the NOS cover three major domains namely, selection of the sample, comparability between groups, and the outcome assessment. Articles are awarded points ranging from zero to nine. Two reviewers conducted the quality assessment and resolved all disagreements by discussion.

### Statistical analysis:

The statistical analysis was conducted on “Review Manager” (RevMan, version 5.3; Nordic Cochrane Centre (Cochrane Collaboration), Copenhagen, Denmark; 2014). We first retrieved all data pertaining to the association between liver enzymes and cognitive impairment or dementia and tabulated it. Data was separated depending upon the outcome i.e. cognitive impairment or dementia and then based on the marker used (ALT, AST, GGT, and AST: ALT). If studies reported different cut-offs, the cut-off closest to the other included articles was used for the analysis. If multiple groups were reported for the same marker, the data was combined using Review Manager to establish a single group for each marker. Then, we pooled data for each marker separately to calculate odds ratio (OR) and 95% CI. A random-effects meta-analysis model was chosen given the baseline differences between the included studies. As the number of studies in each meta-analysis was <10, we did not generate funnel plots for publication bias. Likewise, due to scarce data, subgroup analysis was also not possible. The I^2^ statistic provided the numerical value on heterogeneity between studies with I^2^>50% indicating substantial heterogeneity. Sensitivity analysis was conducted by excluding one study at a time from the meta-analysis and rechecking the results.

## RESULTS

### Search results:

**Fig.1:**
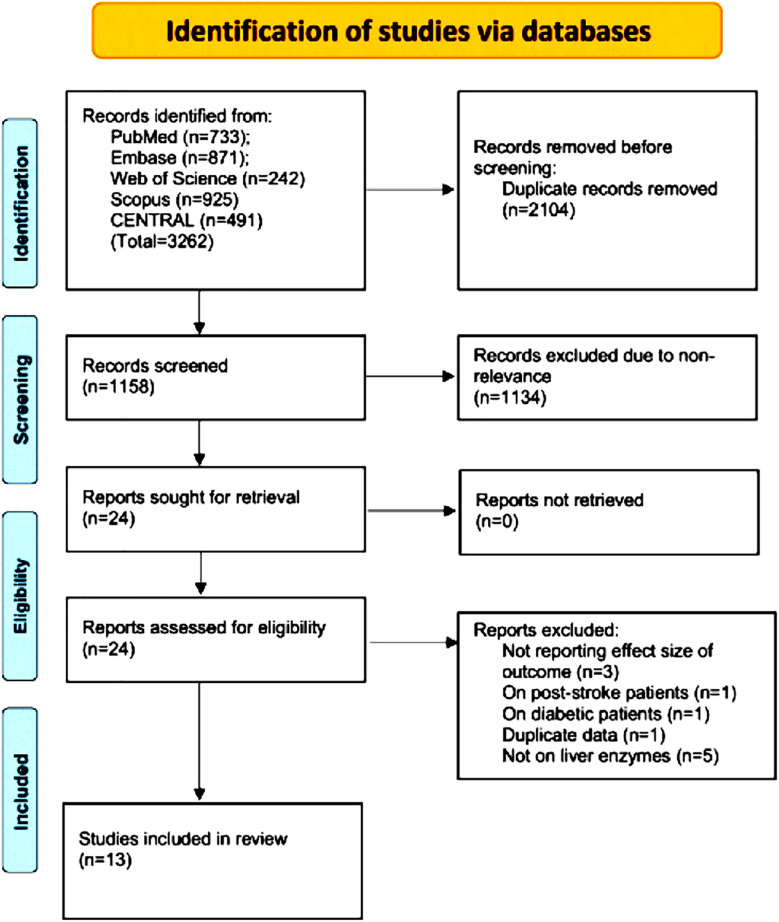
Study flow chart.

The PRISMA flowchart of the study shows the number of articles at every step of the screening process (Fig.1). After finding 3262 articles from the databases, we were able to retrieve 1158 articles following electronic deduplication. These were meticulously screened by the reviewers and 24 were selected for further analysis. Of these 13 articles,^17^-^29^ made it to the final review. Based on kappa (0.95), there was a high inter-reviewer agreement for the selection of studies.

### Details of studies:

The study populations originated from the USA, UK, Finland, Korea and China (Table-I). One study^23^ reported data on three cohorts, of which one was cross-sectional and two were prospective cohorts. Of the remaining 12, three studies were cross-sectional in design. Five were cohort studies of which four were prospective and one was retrospective. The remaining four had a case-control study design. Most studies excluded non-elderly or young adults from the study. Overall, all included studies had a total sample size of 6782347 participants. Eight cohorts reported data on ALT, five on AST, nine on AST: ALT, and seven on GGT. Ten cohorts reported dementia as the outcome and six reported cognitive impairment. The method of assessment of cognitive impairment and dementia varied across studies. All studies reported adjusted outcomes with varying covariates. However, two cohorts did not specify the exact covariates adjusted in the analysis. The quality of studies also varied on NOS. Five cohorts were high quality receiving a score of nine. Three received a score of eight, five got a score of seven, and two were given a score of six.

**Table-I:** Key information of included literature.

Study Location	Type	Excluded participants	Sample size	Age	Males	Marker	Cut-off	Outcome assessment	Adjusted covariates	Follow-up
Zhang 2024 USA	CS	<60 years, missing data	2764	69.2	1357	ALT, AST: ALT, GGT	≥16 U/L, >1, >28	Dementia assessed by CERAD test	Gender, race, age, education level, poverty-income ratio, body mass index, physical activity, smoking, drinking, diabetes, hypertension, stroke, coronary heart disease, liver disease, cholesterol, triglyceride and serum uric acid	-
Huang 2024 UK	PC	<40 years, dementia	367093	57.2	167762	GGT	NR	Dementia identified by ICD codes	Demographic, temporal, socioeconomic, and lifestyle factors	5 years
Gao 2024 UK	PC	<40 years, dementia	346855	NR	NR	ALT AST AST:ALT	NR	Dementia identified by ICD codes	Age, gender, education levels, APOE ε4 carrier status, BMI, smoking and alcohol assumption status, and race	8.65 years
Lu 2023 USA	PC	Stroke/ dementia at baseline, liver disease, significant alcohol consumption, ALT/AST ≥40 U/L	8972	57.1	3992	ALT	≥14 U/L	Dementia assessed by neuropsychological assessments, annual participant or informant contact, and medical record surveillance	Age, sex, race-center, education, and APOE ɛ4 genotype, alcohol use, estimated glomerular filtration rate, body mass index, blood pressure, high density lipoprotein cholesterol, total cholesterol, hypertension, and diabetes.	24.1 years
Han 2023 Korea	CS	Liver cirrhosis, liver cancer, not undergone neuropsychological assessments,	554	75	166	AST, ALT, AST: ALT	NR	Dementia by clinical assessment (AD)	Hypertension, diabetes mellitus, dyslipidemia, and statin use	-
Cushman 2023 USA	CC	Stroke, cognitive impairment	1082	NR	NR	ALT, AST, AST: ALT, GGT	NR	Cognitive impairment assessed by cognitive tests administered by telephone	Age, race, region, sex, education, income, systolic blood pressure, left ventricular hypertrophy, smoking, prebaseline cardiovascular disease, atrial fibrillation, diabetes, and hypertension medication use, alcohol use, body-mass index and physical activity	3.4 years
Li 2022 China	CS	Non-elderly, cognitive impairment	476	74	200	AST: ALT	1.06	Dementia by DSM-IV criteria (AD). Cognitive impairment by clinical diagnosis.	Gender, age, education, smoking, drinking alcohol, drinking tea, hobbies, and fasting blood glucose	-
	PC	Non-elderly, mental illness, physical illness	260	70	117	AST: ALT	1.06	Cognitive impairment by clinical diagnosis.	NR	2 years
	PC	Non-elderly, cognitive impairment	94	72	32	AST: ALT	1.06	Cognitive impairment by clinical diagnosis.	NR	7 years
Han 2022 Korea	CC	Non-elderly, hepatocellular carcinoma, gallbladder cancer, extremes of liver enzymes	580	73	170	AST, ALT, AST: ALT	NR	Dementia by clinical diagnosis based on National Institute on Aging-Alzheimer’s Association workgroups	Education, hypertension, diabetes mellitus, dyslipidemia, body-mass index and APOE ɛ4 carrier status	-
Tang 2021 China	CS	Hepatobiliary and pancreatic diseases, heavy alcoholics, dementia	2943	46	0	GGT	≥22 U/L	Cognitive impairment diagnosed by Montreal Cognitive Assessment score (<26)	Age, body-mass index, education, sleep duration, smoking status, and alcohol consumption, hypertension, hyperlipidemia, diabetes mellitus, serum uric acid, and estrogen level.	-
Lee 2020 Korea	RC	<40 years, dementia, liver cirrhosis, chronic hepatitis, any malignancy, and/or heavy alcohol consumption	6046442	55	NR	GGT	≥23 U/L in men and ≥15 U/L in women	Dementia determined by prescription of anti-dementia drugs	Age, sex, smoking history, alcohol consumption, regular exercise, monthly income, and body mass index, hypertension, diabetes, dyslipidemia, myocardial infarction, stroke, and hospitalized heart failure.	6.3 years
Zhou 2019 China	CC	<60 years, dementia	236	68	90	ALT, AST, GGT	>10.2, >14.6, >22 U/L	Cognitive impairment diagnosed by modified Petersen’s criteria	Age, gender, education level, heart disease, diabetes, hypertension, homocysteine, vitamin B12 and folate levels	-
Nho 2019 USA	CC	NR	1581	74	884	AST, ALT, AST: ALT	NR	Cognitive impairment diagnosed by Mini-Mental State Examination score and dementia by National Institute of Neurological and Communicative Disorders and Stroke-AD and Related Disorders Association criteria	Age, sex, body mass index, and APOE ε4 status.	-
Kunutsor 2016	PC	<40 years, dementia	2415	53.2	2415	GGT	>23 U/L	Dementia diagnosed by neuropsychological testing, and magnetic resonance imaging of the brain	Age, body mass index, systolic blood pressure, history of coronary heart disease, smoking status, history of diabetes, use of medications (antihypertensive agents and lipid-lowering drugs), total cholesterol, high-density lipoprotein cholesterol, alcohol consumption, socioeconomic status, and physical activity, C-reactive protein, incident coronary heart disease	22

AD, Alzheimer's disease; CERAD, Consortium to Establish a Registry for Alzheimer's Disease test; NR, not reported; PC, prospective cohort; RC, retrospective cohort;CS, cross sectional; CC, case-control; ALT, alanine aminotransferase; AST, aspartate aminotransferase; GGT, gamma-glutamyl transpeptidase; U/L, units per litre; ICD, International Classification of Diseases.

**Supplementary Table-I:** Risk of bias analysis.

Study Location	Selection of sample	Comparability	Outcome assessment	NOS score
Zhang 2024 USA	4	2	1	7
Huang 2024 UK	4	2	2	8
Gao 2024 UK	4	2	3	9
Lu 2023 USA	4	2	3	9
Han 2023 Korea	4	2	1	7
Cushman 2023 USA	4	2	-	6
Li 2022 China	4	2	1	7
	4	1	1	6
	4	1	2	7
Han 2022 Korea	4	2	3	9
Tang 2021 China	4	2	1	7
Lee 2020 Korea	4	2	3	9
Zhou 2019 China	4	2	2	8
Nho 2019 USA	4	2	2	8
Kunutsor 2016	4	2	3	9

NOS: Newcastle Ottawa scale.

### Cognitive impairment:

**Fig.2:**
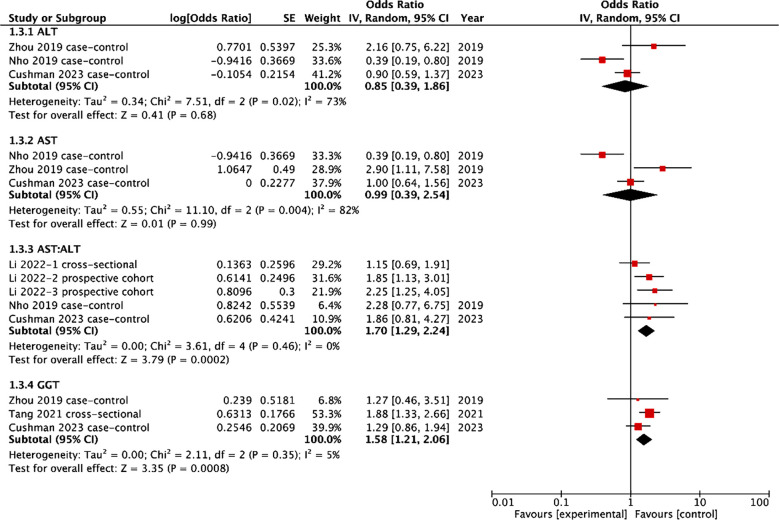
Meta-analysis of the association between liver markers and cognitive impairment.

Results of the meta-analysis examining the link between liver enzyme levels and cognitive impairment are shown in Fig.2. Three studies reported the association between ALT/AST and cognitive impairment. Pooled analysis showed that there was no significant association between ALT levels (OR: 0.85 95% CI: 0.39, 1.86 I^2^=73%) or AST levels (OR: 0.99 95% CI: 0.39, 2.54 I^2^=82%) and risk of cognitive impairment. A total of three studies examined the association between AST: ALT and risk of cognitive impairment. Out of these, one study included three cohorts which were pooled separately. Meta-analysis showed the higher AST: ALT ratio was associated with a statistically significant increased risk of cognitive impairment (OR: 1.70 95% CI: 1.29, 2.24 I^2^=0%). Only three studies reported the association between GGT levels and the risk of cognitive impairment. Pooled analysis showed that higher GGT levels were associated with a statistically significant increased risk of cognitive impairment (OR: 1.58 95% CI: 1.21, 2.06 I^2^=5%). On sensitivity analysis, the results of ALT, AST, and AST:ALT did not change in significance. However, on exclusion of the study of Tang et al,^25^ the results indicated no association between GGT and risk of cognitive impairment (OR: 1.29 95% CI: 0.88, 1.88 I^2^=0%).

### Dementia:

**Fig.3:**
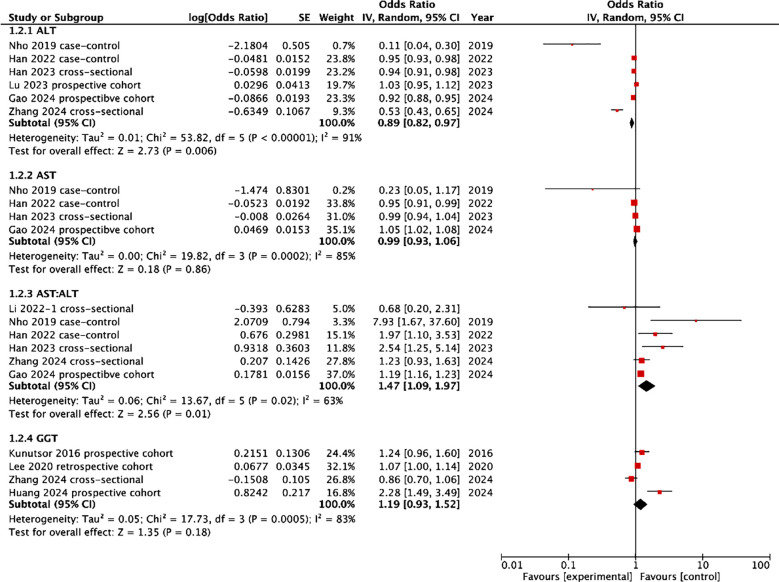
Meta-analysis of the association between liver markers and dementia.

Fig.3 presents the results of the meta-analysis examining the link between liver enzymes and the risk of dementia. A pooled analysis of five studies showed that lower ALT levels were associated with a significant increase in the risk of dementia (OR: 0.89 95% CI: 0.82, 0.97 I^2^=91%). Four studies presented data on the association between AST levels and the risk of dementia. Meta-analysis did not show any statistically significant association between the two (OR: 0.99 95% CI: 0.93, 1.06 I^2^=85%). Six studies reported data on the association between AST: ALT and the risk of dementia. Meta-analysis revealed that higher AST: ALT ratio was associated with a significantly increased risk of dementia (OR: 1.47 95% CI: 1.09, 1.97 I^2^=63%). However, a meta-analysis of four studies did not show any significant association between GGT levels and risk of dementia (OR: 1.19 95% CI: 0.93, 1.52 I^2^=83%). None of the results changed in significance on sensitivity analysis.

## DISCUSSION

The present study showed that evidence on the association between liver markers and risk of cognitive impairment and dementia is limited and conflicting. The pooled analysis showed that there was no significant association between ALT levels or AST levels and risk of cognitive impairment. Meta-analysis showed the higher AST:ALT ratio and higher GGT levels were associated with a statistically significant increased risk of cognitive impairment. Lower ALT levels were associated with a significant increase in the risk of dementia but no significant association was noted with AST or GGT levels.

The increasing age of the world population has driven increased research interest in cognitive aging.^2^,^3^,^30^ Cognitive impairment and dementia are responsible for a significant reduction in productivity and routine activities causing a substantial economic burden on society.^4^ Prevention has been one of the keystones in the management of cognitive aging and dementia. Early identification and alteration of important risk factors can attenuate the risk of cognitive decline and reduce the burden on patients with dementia.^31^ In line with this philosophy, in 2017, the Lancet Commission put forward nine risk factors for the disease namely, chronic isolation, less education, inadequate sleep, reduced physical activity, diabetes, hypertension, smoking, depression, and hearing impairment.^32^ Further, in 2020, the commission included increased alcohol consumption, traumatic brain injury, and air pollution in the “life-course model” of the disease for prevention.^33^ These risk factors account for more than 1/3^rd^ of the global cases of dementia and early identification and alteration can lead to reduced incidence of the disease.^34^

It can be noted that the Lancet Commission has recognized that liver injury due to excessive alcohol consumption can lead to cognitive impairment and dementia.^33^ Indeed, this has been supported by several other studies linking liver diseases with dementia. Parikh et al^35^ in a UK Biobank study of 455,226 participants have found that liver fibrosis is associated with a 1.5 times heightened risk of dementia independent of shared risk factors. Another large study from Israel with 826,578 middle-aged adults and 17 years of median follow-up has noted that patients with inconclusive liver fibrosis have a 9% increased risk of dementia while those with advanced fibrosis have an 18% higher risk of dementia.^36^ A nationwide cohort study from the Korean insurance database involving 4,031,948 participants showed that non-alcoholic fatty liver disease (NAFLD) was independently associated with dementia.^37^

Nevertheless, there have been some controversial results produced by meta-analysis studies. One meta-analysis study^10^ shows that NAFLD is independently associated with dementia while other shows that NAFLD may increase the risk of only cognitive impairment but not dementia.^38^ Furthermore, the change of terminology from NAFLD to metabolic dysfunction-associated fatty liver disease (MAFLD) also seems to have not affected the relationship between the two entities. Research shows that MAFLD patients have reduced cognitive ability with decreased brain volume and cerebral perfusion on magnetic resonance imaging.^39^ Another study has shown that MAFLD is linked with an increased risk of vascular dementia but only the diabetes subtype of MAFLD had an heightened risk of AD.^40^ A Mendelian randomization study has also found genetic causal associations between biopsy-confirmed liver disease and vascular dementia but not with other dementias.^41^

Given such a plethora of evidence linking liver pathology and cognitive impairment/dementia, it can be postulated that liver enzymes which are markers for liver function may act as predictors of the latter. Indeed, on a systematic search of literature we found 13 studies examining such associations and to summarize the available evidence we have performed the first systematic review and meta-analysis of literature. At the outset, it must be stated that the present meta-analysis could include only few studies in each meta-analysis and hence the results must be construed with caution. For better clarity on the associations, we conducted separate analyses for cognitive impairment and dementia and examined each of the biomarkers separately. It was found that high AST:ALT ratio and GGT levels were significantly linked with heightened risk of cognitive impairment. On the other hand, low ALT levels and high AST:ALT ratio were found to increase the risk of dementia. All other associations were found to be non-significant.

AST is predominantly noted in the mitochondria of liver cells while ALT is present in the hepatocyte cytoplasm. Damage to liver cells leads to the release of both enzymes but as the liver function declines further, the AST clearance rate also reduces leading to higher levels as compared to ALT.^42^ Furthermore, AST is also found in other areas like skeletal muscles, the heart, brain while ALT is exclusively a liver-specific enzyme.^11^ The ratio of AST:ALT also called as De Ritis ratio is routinely used to examine liver function and the severity of liver damage.^43^ A high ratio was associated with both cognitive impairment and dementia in our meta-analysis. There could be several possible mechanisms explaining this.

First, both AST and ALT are important for gluconeogenesis in the liver. They act as catalysts in the production of pyruvate from alanine and alpha-ketoglutarate. Lower levels of ALT can reduce pyruvate production which is needed for liver-related gluconeogenesis glucose production. This significantly impacts the peripheral energy metabolism affecting brain metabolism and leading to cognitive decline.^19^ Nho et al^28^ have found that individuals with higher AST: ALT and low ALT have reduced brain glucose metabolism in the orbitofrontal cortex and temporal lobes on imaging indicating attenuated memory and executive function. Secondly, both enzymes have been linked with the synthesis of glutamate which is an excitatory neurotransmitter facilitating synaptic transmissions in hippocampal and cortical areas of the brain. Reduced production of the same can affect memory and cognition function.^44^,^45^ Lastly, Li et al^23^ have found that AST: ALT ratio is associated with altered hippocampus volume. Such a link has also been noted by Naglich et al^46^ between alcohol use indicators and right hippocampal volume. Indeed, alterations in the medial temporal lobe like the hippocampus and amygdala are particularly important in the pathogenesis and diagnosis of AD.^47^

GGT is an enzyme that is intimately connected with the synthesis and metabolism of glutathione and is a marker of hepatic or biliary diseases.^48^ Our analysis found a significant association between GGT and cognitive impairment but not with dementia. The higher risk of cognitive impairment with GGT could be due to an indirect effect. Higher levels of GGT reflect antioxidant inadequacy and increased oxidative stress which is linked with heightened risk of cardiovascular disease.^49^ Indeed, cardiovascular diseases can lead to cognitive decline.^50^ Secondly, it has been suggested that GGT may not be increased only by liver damage but obesity may have a role as well. Higher levels of GGT have been found in obese individuals and obesity has been linked in the pathogenesis of neurodegenerative diseases by means of insulin resistance.^51^,^52^ Nevertheless, these are just potential pathways, and given the limited literature available there is a need for further studies examining the role of GGT in cognitive impairment and dementia.

### Limitations:

Our results should be interpreted against the following limitations. A major drawback is the variation in the study designs available for the analysis. Due to the limited literature, we had to combine cross-sectional, case-control, and cohort studies for the meta-analysis. Of these, only cohort studies can determine a temporal association between liver enzymes and cognitive impairment or dementia. Secondly, despite including 13 studies, few studies could be included in each meta-analysis. This also prevented us from conducting a detailed subgroup analysis or meta-regression based on baseline study characteristics. Thirdly, there was high inter-study heterogeneity, especially in the statistically significant associations between ALT levels, AST:ALT ratio, and dementia.

This could be due to differences in study designs and population, cut-offs of liver enzymes, method of assessment of outcome, and adjusted covariates. Importantly, the present study was unable to assess the exact cut-off of liver enzymes which can be considered to be predictive of cognitive impairment or dementia. The included studies used variable cut-offs or did not report the exact cut-off values. Fourthly, the method of assessment of cognitive impairment and dementia varied significantly amongst studies. While most used clinical assessment, some studies identified cognitive impairment/dementia only by telephonic administered cognitive tests or medical records thereby introducing bias in the results. Lastly, the adjusted covariates in the studies depended on the availability of data. Several confounders could have been missed leading to skewed results.

### Strength of the Review:

Despite these drawbacks, we have presented the first collated evidence on the role of liver enzymes in forecasting the risk of cognitive impairment/dementia. We used only adjusted effect sizes to limit errors due to confounding and performed a separate meta-analysis for each liver biomarker. Liver markers like AST, ALT, GGT are routinely available in all healthcare setups and hence, have potential to be biomarkers for cognitive impairment/dementia in clinical practice. While these results may not be immediately applicable in routine practice, we believe that the evidence generated in our review will prompt further research in establishing the utility of liver biomarkers in predicting the risk of cognitive impairment/dementia. Further cohort studies with long-term follow-up utilizing similar cut-offs of these biomarkers and examining their accuracy for predicting cognitive impairment and dementia are needed to generate better evidence.

## CONCLUSIONS

Preliminary evidence indicates that liver biomarkers may have a role in predicting the risk of cognitive impairment/dementia. A high AST: ALT ratio may be linked with an increased risk of cognitive impairment and dementia. Likewise, high GGT and low ALT levels seem to be associated with increased risk of cognitive impairment and dementia respectively. Future cohort studies with long follow-up periods taking into account all confounding factors and using specific cut-offs of liver enzymes are needed to provide better quality of evidence.

### Authors’ contributions:

**YZ:** Study design. Literature search, and manuscript writing.

**YZ and LL:** Data collection, data analysis and interpretation. Critical Review.

**YZ:** Manuscript revision and validation and is responsible for the integrity of the study.

All authors have read and approved the final manuscript.
